# Molecular, Metabolic and Inflammatory Patterns Involved in Pathogenesis of Anderson-Fabry Disease

**DOI:** 10.3390/cells15050443

**Published:** 2026-02-28

**Authors:** Irene Simonetta, Irene Baglio, Antonino Tuttolomondo

**Affiliations:** Internal Medicine and Stroke Care Ward, Regional Reference Center for Diagnosis and Treatment of Anderson-Fabry Disease, Department of Health Promotion, Maternal and Child Health, Internal Medicine and Specialty Excellence “G. D’Alessandro” (PROMISE), University of Palermo, Piazza delle Cliniche n.2, 90127 Palermo, Italybruno.tuttolomondo@unipa.it (A.T.)

**Keywords:** Fabry disease, lysosomal dysfunction, metabolic inflammation, TLR4/NF-κB pathway, mitochondrial impairment

## Abstract

Anderson–Fabry disease (FD) is an X-linked lysosomal storage disorder caused by pathogenic variants in the GLA gene, resulting in deficient α-galactosidase A activity and progressive accumulation of globotriaosylceramide (Gb3) and its derivative lyso-Gb3 within lysosomes. Beyond substrate storage, FD involves a complex interplay of molecular, metabolic, and inflammatory disturbances that collectively drive multisystemic damage. It seems that Gb3 accumulation impairs autophagic flux, promotes mitochondrial dysfunction, and triggers endoplasmic reticulum stress, leading to oxidative imbalance and bioenergetic failure. Concurrently, activation of innate immune pathways, particularly the TLR4/NF-κB axis, induces pro-inflammatory cytokine release and endothelial dysfunction, while complement activation and adaptive immune responses contribute to chronic inflammation and fibrosis. These mechanisms define a sustained state of “metaflammation,” linking lysosomal dysfunction to systemic inflammation. Understanding this molecular cross-talk provides a rationale for identifying novel biomarkers and designing therapies that go beyond enzymatic correction, including chaperone therapy, substrate reduction, and gene-based or anti-inflammatory approaches. A deeper comprehension of these interconnected patterns may guide the development of precision medicine strategies aimed at improving long-term outcomes in Fabry disease.

## 1. Introduction

Anderson–Fabry disease (FD) is an X-linked lysosomal storage disorder caused by pathogenic variants in the *GLA* gene resulting in reduced or absent α-galactosidase A activity, and consequent progressive accumulation of globotriaosylceramide (Gb3) and its deacylated derivative globotriaosylsphingosine (lyso-Gb3) in multiple cell types and tissues, also causing neurogenic inflammation and alterations of the peripheral nervous system in host defense and immunopathology [1].

Clinically, Fabry disease typically presents early in life with neuropathic pain, angiokeratomas and hypohidrosis, and progressively leads to renal impairment, cardiomyopathy, cerebrovascular complications and reduced life expectancy. This clinical course reflects the systemic nature of the disorder and highlights the need for a deeper understanding of the mechanisms that drive disease progression, beyond symptomatic organ involvement [2].

From a broader biological standpoint, Fabry disease illustrates how a single-gene defect affecting a lysosomal enzyme can give rise to a complex cascade of secondary alterations involving cellular metabolism, mitochondrial function and immune activation. These downstream processes extend the classical concept of lysosomal storage disorders toward a more integrated view that overlaps with mechanisms observed in chronic metabolic and inflammatory diseases [2]. Although several recent reviews have explored Fabry disease beyond lysosomal substrate accumulation, the present work focuses on bringing together molecular, metabolic and immune–inflammatory alterations within a coherent pathogenetic perspective. In this context, Fabry disease is discussed as a model of inherited metabolic inflammation (“metaflammation”), primarily as an interpretative framework to support biomarker stratification and the identification of biologically grounded therapeutic targets.

Historically, the pathogenesis of Fabry disease was largely interpreted as a direct consequence of intracellular globotriaosylceramide accumulation, with lysosomal storage assumed to cause mechanical and functional disruption of cells and tissues. While this concept remains fundamental, it does not fully account for the wide phenotypic variability observed in clinical practice, including organ-specific vulnerability—particularly affecting the heart and kidneys—sex-related differences, and the heterogeneous response to enzyme replacement therapy [3]. These limitations have prompted a shift in research focus toward mechanistic studies investigating how lysosomal dysfunction initiates secondary disturbances in cellular metabolism and bioenergetic homeostasis, which may contribute independently to disease progression (Table 1).

Accumulating evidence indicates that the build-up of Gb3 and lyso-Gb3 interferes with intracellular quality-control mechanisms, particularly autophagy and mitophagy. As a consequence, damaged mitochondria tend to persist, reactive oxygen species increase, and cellular energy production becomes progressively inefficient [4,5]. Importantly, experimental and translational studies suggest that these metabolic and mitochondrial abnormalities may arise relatively early in the disease course and, in some settings, even before extensive substrate accumulation is detectable. This observation raises the possibility that bioenergetic dysfunction contributes independently to cellular stress and vulnerability in Fabry disease [6].

Alongside metabolic alterations, inflammation is now viewed as an active component of disease progression rather than a purely secondary response to cellular injury. Both experimental models and patient-based studies indicate that glycosphingolipid accumulation can engage innate immune signaling pathways, thereby promoting endothelial activation, cytokine release and recruitment of immune cells [4,7]. Consistently, inflammatory profiles have been shown to correlate with disease severity and organ involvement, and in several cases persist despite enzyme replacement therapy. This persistence suggests that immune–metabolic feedback loops may become partially self-maintaining over time [8].

Whether inflammatory activation in Fabry disease remains tightly linked to substrate burden or gradually evolves into a more autonomous pathological axis remains an open question. Notably, the progression of inflammatory and fibrotic changes even after delayed substrate reduction supports the notion that immune mechanisms, particularly humoral pathways such as complement activation, may act as independent drivers of vascular and organ damage, extending disease pathogenesis beyond lysosomal storage alone [9,10].

From a diagnostic standpoint, lyso-Gb3 remains the most reliable biochemical marker for Fabry disease, particularly in male patients. Nevertheless, its levels frequently fail to fully normalize during treatment and appear to reflect only part of the underlying disease activity, providing limited insight into ongoing inflammatory or mitochondrial dysfunction [4,5]. For this reason, growing interest has focused on complementary biomarker approaches. Panels combining inflammatory, endothelial, and metabolic markers, together with multiomic strategies, may offer a more integrated view of disease dynamics by capturing alterations in mitochondrial function, endoplasmic reticulum stress, and immune signaling pathways [6].

Similarly, therapeutic perspectives have evolved alongside the recognition that Fabry disease extends beyond isolated lysosomal dysfunction. While enzyme replacement therapy and pharmacological chaperones remain the cornerstone of current management, their reduced effectiveness in later disease stages has underscored the need for additional strategies. Emerging approaches, including substrate reduction, gene- and RNA-based therapies, mitochondrial-protective compounds, and immune-modulating interventions, are being explored to target secondary pathogenic mechanisms that are not adequately addressed by enzyme correction alone. In this context, the early identification of metabolic and inflammatory signatures may prove valuable for patient stratification and for guiding the use of adjunctive therapies alongside standard enzyme-based treatment [6,7].

### Methodological Approach and Aim of the Review

This narrative review is based on a structured survey of the literature conducted using the PubMed and Scopus databases, focusing on peer-reviewed articles published over the past decade to capture the most up-to-date developments in the field. Particular attention was given to studies with clear methodological design and relevance to Fabry disease pathophysiology.

When available, priority was assigned to clinical studies involving larger or well-characterized patient cohorts, as well as to experimental investigations employing established and biologically meaningful animal models capable of reproducing key molecular and metabolic features of the disease. Each study was evaluated with regard to scientific rigor, methodological quality, and its contribution to understanding molecular, metabolic, and inflammatory mechanisms involved in organ damage. This selective and integrative approach was adopted to reduce potential selection bias while ensuring a balanced overview of both mechanistic and translational evidence.

Several recent reviews have already expanded the classical concept of Fabry disease as a purely lysosomal storage disorder, and these works constitute important reference points for the present analysis. In particular, Bertoldi et al. emphasized secondary biochemical alterations downstream of lysosomal dysfunction, with a focus on their therapeutic implications, whereas Biddeci et al. centered on inflammatory mechanisms and their role in clinical progression, organ involvement, and disease-related complications.

In contrast, the present review adopts a complementary and more mechanistically oriented perspective. Rather than prioritizing therapeutic translation or clinical manifestations, the focus is placed on a detailed and integrated examination of the molecular, metabolic, inflammatory, and immune-mediated processes underlying Fabry disease pathogenesis—many of which remain only partially defined. By dissecting the biochemical and bioenergetic links between lysosomal dysfunction, mitochondrial impairment, and chronic immune activation, this work aims to provide a coherent mechanistic framework that may inform future developments in biomarker stratification and precision-medicine approaches. References to organ damage and therapeutic strategies are therefore intentionally secondary and are used primarily to contextualize the biological relevance of the mechanistic networks discussed.

Against this background, the objective of this review is to synthesize and critically assess current evidence on molecular, metabolic, and inflammatory pathways involved in Fabry disease; to explore their points of convergence and interaction; to highlight areas of controversy and unresolved questions, such as the degree of inflammatory autonomy from substrate accumulation and the temporal development of fibrosis, and to discuss how these insights may guide emerging biomarker research and therapeutic innovation. By framing Fabry disease within this integrated mechanistic context, the review seeks to support precision-medicine strategies that move beyond enzyme replacement alone and better reflect the biological complexity of the disease.

## 2. Molecular Basis of Fabry Disease

The enzymatic defect of alfa galactosidase A prevents the degradation of neutral glycosphingolipids, primarily globotriaosylceramide (Gb3) and its derivative globotriaosylsphingosine (lyso-Gb3), resulting in their progressive intracellular accumulation across multiple cell types, including endothelial cells, cardiomyocytes, podocytes, neurons, fibroblasts, and smooth muscle cells [3]. The progressive storage of Gb3 within lysosomes disrupts their function by altering luminal pH, impairing the activity of hydrolytic enzymes, and interfering with vesicular trafficking and membrane recycling [4]. Lyso-Gb3, although present at lower concentrations than Gb3, has been shown to exert potent biological effects, including stimulation of smooth muscle cell proliferation, podocyte injury, and activation of pro-hypertrophic signaling pathways in cardiomyocytes [1]. Studies have demonstrated that lyso-Gb3 also acts as a pro-inflammatory mediator, inducing secretion of cytokines such as IL-6, TNF-α, and MCP-1, which contribute to early renal and vascular injury [5]. More than 900 pathogenic variants in the GLA gene have been identified, producing varying levels of residual enzyme activity and resulting in a wide spectrum of clinical phenotypes, from classic early-onset forms to later-onset, organ-selective variants [6].

The accumulation of Gb3 and lyso-Gb3 triggers profound alterations in several interconnected cellular pathways. A central element of these disturbances is the impairment of autophagy, which results from defective lysosomal clearance and leads to the accumulation of autophagosomes and dysfunctional mitochondria. This disruption is particularly evident in cardiomyocytes and podocytes, where impaired autophagy contributes to hypertrophy, proteinuria, and organ-specific damage. Another important aspect of the molecular basis of Fabry disease is the induction of endoplasmic reticulum (ER) stress caused by misfolded α-galactosidase A proteins retained within the ER. These misfolded proteins activate the unfolded protein response (UPR), which aims to restore protein homeostasis but can lead to apoptosis and inflammation when chronically activated [7]. Additionally, Gb3 accumulation alters membrane architecture and facilitates the formation of lipid microdomains that interfere with signaling pathways involving growth factors, ion channels, and mechanosensitive receptors. This contributes to vascular dysfunction and hypertrophy, two major hallmarks of the disease [8]. The progressive intracellular accumulation of Gb3 also interferes with mitochondrial function, reducing ATP production, impairing oxidative phosphorylation, and enhancing the generation of reactive oxygen species (ROS). These mitochondrial changes contribute to energetic failure, cellular dysfunction, and apoptotic signaling, particularly in high-energy tissues such as myocardium, kidney, and the peripheral nervous system [9].

ROS overproduction further amplifies the cellular stress response by activating redox-sensitive transcription factors, including NF-κB and AP-1, promoting pro-inflammatory cytokine release and linking oxidative stress to chronic inflammation in Fabry disease [10]. Additionally, alterations in mitochondrial dynamics, including impaired fusion, excessive fission, and reduced mitophagy, have been observed in experimental models of Fabry disease. These abnormalities exacerbate mitochondrial fragmentation, bioenergetic insufficiency, and susceptibility to stress-induced cell death. In addition to impaired autophagy and mitochondrial dysfunction, Gb3 and lyso-Gb3 accumulation also affects lysosomal–endosomal trafficking, altering the internalization, recycling, and degradation of membrane receptors and transporters. This contributes to abnormal cell signaling, dysregulated ion homeostasis, and endothelial dysfunction, all of which are key hallmarks of Fabry disease pathophysiology [11]. Moreover, the glycosphingolipid accumulation modifies the lipid composition of the plasma membrane, promoting the formation of altered lipid rafts that interfere with receptor clustering, mechanotransduction, and growth factor signaling. These changes have been associated with vascular instability, increased sensitivity to shear stress, and progressive cardiomyopathic remodeling [12]. Finally, alterations in intracellular trafficking pathways influence the turnover and localization of key proteins involved in calcium handling, redox balance, and stress signaling. This further disrupts cellular homeostasis and contributes to the progressive dysfunction of organs primarily affected by the disease, such as the heart, kidneys, and nervous system. Another fundamental molecular component of Fabry disease is the persistent accumulation of lyso-Gb3, which exerts biological effects beyond simple substrate storage. Lyso-Gb3 has been shown to act as a potent signaling lipid, capable of inducing proliferation in vascular smooth muscle cells, altering podocyte architecture, and promoting pro-fibrotic transcriptional programs that contribute to progressive organ damage. In addition to its proliferative effects, lyso-Gb3 stimulates the expression of inflammatory cytokines and chemokines, linking substrate accumulation to early immune activation and creating a pro-inflammatory microenvironment in affected tissues such as the kidney and myocardium [13]. Furthermore, lyso-Gb3 contributes to extracellular matrix remodeling by activating fibroblasts and promoting the deposition of collagen and other matrix proteins, accelerating fibrotic processes. This mechanism plays a central role in the development of cardiac and renal fibrosis in Fabry patients, even in the presence of enzyme replacement therapy [14].

## 3. Metabolic Alterations and Organelle Dysfunction

Fabry disease is associated with marked disturbances in cellular bioenergetics, largely driven by the intracellular accumulation of Gb3 and lyso-Gb3 and their impact on mitochondrial function [15]. Experimental studies consistently show a progressive impairment of mitochondrial respiration, characterized by defects in oxidative phosphorylation, reduced ATP generation, and increased production of reactive oxygen species (ROS) [12]. These alterations are especially evident in cells with high energetic requirements—such as cardiomyocytes, podocytes, and neurons—where mitochondrial dysfunction contributes directly to tissue-specific vulnerability and functional decline [16,17]. The persistence of damaged mitochondria further amplifies metabolic stress and facilitates activation of pro-apoptotic signaling pathways [18].

Alongside mitochondrial impairment, disruption of autophagy represents a central component of metabolic remodeling in Fabry disease. Accumulated Gb3 interferes with the fusion of autophagosomes and lysosomes, resulting in defective autophagic flux and progressive intracellular accumulation of undegraded material [19]. Inefficient clearance of dysfunctional mitochondria not only worsens oxidative stress but also perturbs lipid turnover, thereby aggravating intracellular energy imbalance. Over time, impaired autophagy establishes a self-reinforcing loop in which mitochondrial dysfunction and ROS production perpetuate one another [20].

Endoplasmic reticulum (ER) stress constitutes an additional layer of metabolic dysregulation. The buildup of misfolded or undegraded proteins within the ER activates the unfolded protein response (UPR), a compensatory mechanism that becomes maladaptive when chronically engaged. Sustained UPR activation leads to reduced protein synthesis, altered calcium homeostasis, and initiation of apoptotic signaling cascades [21]. Importantly, ER stress also affects mitochondrial function through disruption of mitochondria-associated membranes (MAMs), specialized sites that coordinate calcium transfer, lipid exchange, and mitochondrial dynamics. Structural and functional alterations at these interfaces further compromise cellular homeostasis and increase susceptibility to metabolic stress [22].

An additional dimension of metabolic dysregulation in Fabry disease involves alterations in mTOR signaling, a key pathway regulating nutrient sensing, energy balance, and cellular growth. Lysosomal dysfunction induced by Gb3 accumulation can perturb mTORC1 activity, reducing the capacity of cells to adapt appropriately to changes in nutrient availability and energetic demand. This imbalance favors a metabolic shift toward glycolysis, loss of metabolic flexibility, and abnormal regulation of cell growth. Together with mitochondrial and endoplasmic reticulum stress, these changes contribute to a broad remodeling of cellular metabolism that may precede overt organ dysfunction, underscoring the central role of bioenergetic failure in Fabry disease pathophysiology.

Altered communication between intracellular organelles represents a further critical aspect of this remodeling process. Disruption of ER–mitochondria crosstalk interferes with calcium homeostasis, lipid transfer, and stress-response signaling, ultimately compromising mitochondrial respiration and increasing vulnerability to oxidative injury [23]. In parallel, defective lysosomal function limits the efficient removal of damaged mitochondria through mitophagy, leading to the persistence of fragmented, ROS-producing organelles. This accumulation exacerbates redox imbalance and sustains chronic metabolic stress, particularly in energetically demanding cells such as cardiomyocytes and renal epithelial cells [24].

Disturbances in lipid metabolism further contribute to disease progression. Gb3 accumulation alters the synthesis and distribution of membrane lipids, affecting the organization of lipid rafts and, consequently, the spatial arrangement and function of membrane-associated receptors. These structural changes influence growth factor signaling, mechanotransduction pathways, and endothelial responses to hemodynamic stress [25]. Collectively, these metabolic and structural alterations define a complex landscape of organelle dysfunction that may drive disease progression well before irreversible fibrotic changes become clinically apparent.

## 4. Inflammatory Mechanisms

Inflammation plays a central and early role in the progression of Fabry disease, emerging not simply as a secondary response to substrate accumulation but as a primary driver of tissue injury. Lyso-Gb3 acts as a potent pro-inflammatory lipid mediator, engaging Toll-like receptor 4 (TLR4) and triggering downstream NF-κB activation, which promotes transcription of cytokines such as TNF-α, IL-1β, and IL-6 [15]. This TLR4-mediated inflammatory signaling contributes to endothelial activation, increased vascular permeability, and early renal and cardiac involvement. Studies demonstrate that lyso-Gb3 exposure increases the expression of adhesion molecules (VCAM-1, ICAM-1) on endothelial cells, facilitating leukocyte recruitment and chronic inflammatory infiltration [16]. Activation of the complement system represents another key contributor to inflammation in Fabry disease. Elevated plasma levels of complement fragments such as C3a and C5a have been observed in Fabry patients and correlate with endothelial dysfunction, microvascular injury, and progression of renal disease. C5a, in particular, acts as a potent anaphylatoxin that promotes chemotaxis, cytokine release, and oxidative burst in immune cells [17]. Complement deposition within renal tissue has been documented in Fabry nephropathy, where C5b-9 membrane attack complex contributes to podocyte injury and proteinuria, linking complement dysregulation to glomerular dysfunction [18].

In Fabry disease, growing evidence points to disruption of the endothelial glycocalyx as an additional mechanism linking chronic inflammation to vascular dysfunction. Clinical studies have shown that Fabry patients exhibit reduced glycocalyx thickness and impaired microvascular perfusion, alterations that may partially improve following disease-specific treatment with enzyme replacement therapy or migalastat [19]. Mechanistically, glycocalyx degradation in Fabry disease appears to be driven by inflammatory signaling pathways, particularly those involving angiopoietin-2 and increased heparanase activity. This process leads to exposure of endothelial adhesion molecules, enhanced vascular permeability, and facilitated leukocyte adhesion and transmigration across the vascular wall. When persistent, glycocalyx damage may sustain endothelial activation and promote chronic microvascular inflammation, thereby contributing to the development of Fabry-related vasculopathy.

Importantly, experimental observations suggest that glycocalyx impairment is potentially reversible. Anti-inflammatory interventions, heparin administration, and pharmacological activation of the Tie2 pathway have been shown to restore glycocalyx integrity, underscoring its dynamic nature. These findings highlight the endothelial glycocalyx as a modifiable interface between metabolic inflammation and vascular injury in Fabry disease [19].

The adaptive immune system is also affected in Fabry disease. Patients exhibit alterations in T-cell activation, increased circulating CD4+ and CD8+ T cells, and enhanced B-cell responses. Immune dysregulation has been reported even in patients receiving enzyme replacement therapy, indicating persistent immunological activation despite partial metabolic correction [19]. Additionally, misfolded α-galactosidase A variants retained in the ER can serve as neo-antigens, promoting the development of neutralizing antibodies in some patients after enzyme replacement therapy, further amplifying inflammation and contributing to variable treatment response [20].

Inflammation and oxidative stress form a mutually reinforcing cycle in Fabry disease. Mitochondrial-derived ROS amplify inflammatory signaling, while cytokines such as TNF-α and IL-6 impair mitochondrial respiration and promote ROS generation. This bidirectional relationship perpetuates tissue damage and accelerates progression toward fibrosis [21]. This chronic inflammatory state contributes to fibrosis in multiple organs, including the heart, kidney, and vascular tissues. Fibrotic remodeling persists even in patients with improved biochemical markers following enzyme replacement therapy, suggesting that inflammation represents a therapeutic target independent of substrate reduction [22].

## 5. Interconnection Between Molecular, Metabolic, and Inflammatory Patterns

### 5.1. Lysosomal Dysfunction and Metabolic Reprogramming

Anderson–Fabry disease (FD) is no longer viewed as a purely lysosomal storage disorder but as a multisystemic metabolic disease characterized by profound molecular and bioenergetic perturbations. The deficiency of α-galactosidase A leads to the accumulation of globotriaosylceramide (Gb3) and its deacylated derivative lyso-Gb3, which act not only as inert storage compounds but also as bioactive lipids interfering with lysosomal–mitochondrial communication, endoplasmic reticulum (ER) integrity, and autophagic flux [24].

Recent studies demonstrate that substrate overload impairs lysosomal acidification, disturbs autophagosome–lysosome fusion, and causes secondary accumulation of autophagic vacuoles and dysfunctional mitochondria [2]. These defects generate a chronic state of bioenergetic failure, marked by decreased ATP synthesis, altered NAD+/NADH ratios, and increased production of reactive oxygen species (ROS). Mitochondrial dysfunction is accompanied by downregulation of oxidative phosphorylation complexes, especially complexes I and IV, and suppression of peroxisome proliferator-activated receptor gamma coactivator 1-alpha (PGC-1α), a master regulator of mitochondrial biogenesis [23].

This metabolic stress activates the mTOR/AMPK signaling axis, which senses nutrient and energy status. Excess Gb3 inhibits AMPK phosphorylation and constitutively activates mTORC1, resulting in defective autophagy, increased protein synthesis, and accumulation of damaged organelles [22,24]. Moreover, ER stress and the unfolded-protein response (UPR) contribute to the release of inflammatory mediators and apoptotic factors. Misfolded glycoproteins within the ER activate PERK, ATF6, and IRE1α, inducing CHOP-mediated apoptosis and the production of IL-6 and CCL2 [16,24,26].

Biddeci et al. and Feriozzi et al. have emphasized that these metabolic perturbations are not merely intracellular phenomena but extend to systemic metabolic rewiring, including lipidome alterations, oxidative imbalance, and abnormal redox signaling [16,23]. In both plasma and tissue biopsies from FD patients, elevated advanced oxidation protein products (AOPP), decreased thiol groups, and reduced ferric-reducing antioxidant power (FRAP) reflect an exhausted antioxidant system, as shown by Simoncini et al. Notably, oxidative stress is detectable even in treatment-naïve patients with normal lyso-Gb3 levels, indicating that redox imbalance may precede overt substrate accumulation [26]. Early oxidative stress has been reported in some cohorts and remains a hypothesis needing confirmation in larger longitudinal studies. These findings support the hypothesis that mitochondrial and ER stress act as early amplifiers of the metabolic defect, bridging the gap between molecular dysfunction and inflammation.

**Figure 1:**
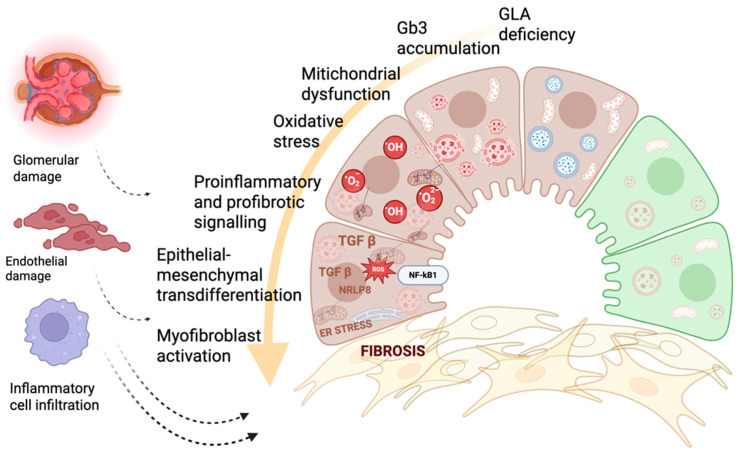
Integrated molecular, metabolic, and inflammatory mechanisms driving organ damage and fibrosis in Fabry disease. Schematic representation illustrating how α-galactosidase A (GLA) deficiency leads to progressive lysosomal accumulation of globotriaosylceramide (Gb3) and related glycosphingolipids, initiating a cascade of interconnected pathogenic events. Substrate overload impairs lysosomal function, disrupts autophagy, and induces mitochondrial dysfunction with excessive production of reactive oxygen species (ROS). Oxidative stress, endoplasmic reticulum (ER) stress, and activation of innate immune pathways (TLR4/NF-κB and NLRP3 inflammasome) converge to amplify proinflammatory and profibrotic signaling, including TGF-β–mediated transcriptional programs. These pathways promote epithelial–mesenchymal transdifferentiation and myofibroblast activation, ultimately driving extracellular matrix deposition and tissue fibrosis. The downstream consequences include endothelial dysfunction, glomerular structural injury, and inflammatory cell infiltration, consistent with the multi-organ progression observed in Fabry disease. This figure highlights the tight interplay between molecular, metabolic, and inflammatory perturbations underlying irreversible organ damage despite enzyme-focused therapies.

In summary, lysosomal dysfunction in FD triggers a cascade of metabolic reprogramming involving autophagy impairment, mitochondrial injury, oxidative stress, and UPR activation. These processes not only contribute to tissue damage but also serve as molecular signals that recruit the innate immune system, setting the stage for a self-perpetuating inflammatory response. Figure 1 provides a simplified schematic overview of the molecular, metabolic, and inflammatory mechanisms underlying organ damage in Fabry disease.

Emerging evidence indicates that chronic inflammation is not unique to Fabry disease but represents a shared pathogenic amplifier across several genetic neuromuscular and metabolic disorders. In Duchenne muscular dystrophy, persistent activation of innate and adaptive immune pathways contributes to muscle degeneration, impaired regeneration, and fibrotic remodeling, with NF-κB–driven signaling and macrophage polarization playing central roles; accordingly, anti-inflammatory and immunomodulatory strategies have been explored as adjunctive therapeutic approaches [27,28].

Similarly, in Pompe disease and other lysosomal storage disorders with prominent neuromuscular involvement, lysosomal dysfunction and impaired autophagy can promote secondary mitochondrial stress and inflammatory signaling, suggesting convergence with the lysosome–mitochondria–inflammation axis described in Fabry disease [29].

Primary mitochondrial disorders also exhibit immune–metabolic crosstalk, whereby mitochondrial damage-associated molecular patterns, including mitochondrial DNA and cardiolipin fragments, activate innate immune pathways such as TLR9 and inflammasome signaling, sustaining chronic low-grade inflammation [30,31].

Collectively, these parallels support the concept that metabolic stress and organelle dysfunction can generate self-perpetuating inflammatory circuits (“metaflammation”) across genetically determined neuromuscular diseases. While disease-specific mechanisms and therapeutic windows differ, this comparative perspective reinforces the rationale for targeting immune–metabolic pathways—such as NF-κB, inflammasome activation, complement, and redox signaling—as potential adjunctive strategies in Fabry disease.

### 5.2. Metabolic–Inflammatory Crosstalk and Immune Activation

The transition from isolated metabolic stress to chronic inflammation represents a pivotal step in Fabry disease (FD) pathogenesis. Accumulated lyso-Gb3 and related glycosphingolipids act as danger-associated molecular patterns (DAMPs), activating pattern-recognition receptors such as Toll-like receptor 4 (TLR4) and downstream NF-κB signaling, with subsequent induction of pro-inflammatory cytokines including TNF-α, IL-1β, and IL-6 [3,32]. This metabolic–inflammatory coupling sustains a state of chronic “metaflammation,” in which metabolic dysfunction and immune activation reinforce each other through self-amplifying loops [3].

Gb3 accumulation within endothelial and smooth-muscle cells enhances NADPH oxidase activity, leading to excessive reactive oxygen species (ROS) production. Oxidative stress further potentiates NF-κB signaling and promotes activation of the NLRP3 inflammasome, resulting in maturation of IL-1β and IL-18 and contributing to vascular inflammation and fibrosis [33]. In parallel, pro-inflammatory cytokines directly impair mitochondrial respiration, reduce ATP generation, and exacerbate oxidative stress, thereby perpetuating mitochondrial dysfunction [34]. Lyso-Gb3 thus represents a key molecular link between substrate storage, inflammation, and fibrotic remodeling by promoting cytokine release, fibroblast activation, and extracellular matrix deposition [19].

Organelle stress further amplifies immune activation. Endoplasmic reticulum stress induced by misfolded α-galactosidase A variants activates the unfolded protein response, which enhances cytokine production and interferes with mitochondrial function, linking protein misfolding to inflammation even in late-onset phenotypes [35]. Together, these mechanisms indicate that FD pathogenesis extends beyond lysosomal storage to involve an interconnected network of metabolic, molecular, and inflammatory disturbances that drive disease progression before overt organ damage becomes clinically apparent.

Inflammatory dysregulation in FD also involves humoral immune pathways. Complement activation, particularly through the alternative and lectin pathways, has been associated with endothelial injury, microvascular remodeling, and organ involvement. Longitudinal immunogenetic analyses have demonstrated persistent complement activation and elevated cytokine levels despite long-term enzyme replacement therapy, with genetic variants in TLR4, IL6, and TNFA modulating inflammatory profiles and clinical severity [36]. Activation of C5a and the membrane attack complex contributes to oxidative stress, endothelial injury, and renal fibrosis, highlighting complement as an active driver of disease progression rather than a mere downstream consequence of storage [33,37].

Finally, mitochondrial dysfunction itself acts as a pro-inflammatory signal. Damaged mitochondria release mitochondrial DNA and cardiolipin fragments that activate TLR9 and NLRP3, further sustaining inflammation and fibrosis [38,39]. Consistently, alterations in adaptive immunity—characterized by increased activated CD8^+^ T cells and reduced regulatory T cells—have been reported, supporting the concept of FD as a prototypical disorder of metabolic inflammation in which lysosomal, mitochondrial, and immune dysfunction are tightly interconnected [40].

## 6. Biomarkers of Pathogenesis and Disease Progression

The identification of reliable biomarkers in Anderson–Fabry disease (FD) remains a central challenge. Due to the disease’s long asymptomatic latency, clinical heterogeneity, and variable therapeutic responsiveness, early and accurate detection of biochemical, metabolic, or inflammatory alterations is pivotal for improving patient stratification, therapeutic timing, and outcome prediction [32,33]. Over the past decade, biomarker research in FD has shifted from single-analyte approaches toward multiparametric, *omics*-driven signatures that reflect the complex interplay between lysosomal dysfunction, metabolic stress, and chronic inflammation [23]. As shown in Table 2. emerging biomarkers in Fabry disease are categorized according to their type, biological source, analytical method, and clinical significance

### 6.1. Classical Biochemical Markers: Gb3 and Lyso-Gb3

Traditionally, plasma or urinary globotriaosylceramide (Gb3) quantification served as a biochemical hallmark of FD. However, its diagnostic sensitivity is limited—particularly in late-onset forms and heterozygous females—due to overlapping values with healthy individuals and poor correlation with clinical severity [32,38]. The subsequent discovery of the deacylated derivative globotriaosylsphingosine (lyso-Gb3) represented a major advance. Lyso-Gb3 is more soluble and diffusible than Gb3 and accumulates systemically in plasma, urine, and tissues. It is now considered the reference biochemical biomarker for diagnosis, screening, and therapeutic monitoring [32,33].

Elevated plasma lyso-Gb3 concentrations correlate with *GLA* genotype, disease phenotype, and sex. Classic hemizygous males typically exhibit markedly increased values (>100 ng/mL), late-onset variants show moderate elevations, and heterozygous females present variable levels depending on X-chromosome inactivation [28,33]. The ratio of α-galactosidase A activity to lyso-Gb3 concentration in dried-blood spots improves diagnostic accuracy, particularly in females [44]. Moreover, cumulative exposure to lyso-Gb3 (product of concentration × age) appears to better reflect total metabolic burden and may serve as a composite indicator of long-term risk [32].

Beyond its diagnostic role, lyso-Gb3 is mechanistically relevant. In vitro, it induces endothelial activation, smooth-muscle proliferation, oxidative stress, and TLR4/NF-κB-mediated cytokine release, thereby bridging lysosomal dysfunction and inflammatory signaling [10,45]. Clinically, lyso-Gb3 correlates with left-ventricular mass index (LVMI), Mainz Severity Score Index (MSSI), and the extent of white-matter lesions in MRI studies, although variability across cohorts limits its prognostic precision. Its decline during enzyme replacement therapy (ERT) is rapid in classic males but often incomplete in females or advanced disease, indicating residual cellular storage or ongoing inflammation [46].

### 6.2. Lyso-Gb3 Analogues and Glycosphingolipid Isoforms

Recent advances in high-resolution mass spectrometry have revealed multiple lyso-Gb3 analogues differing in sphingoid-base length and saturation (e.g., −28 Da, −12 Da, +14 Da, +16 Da, +34 Da, +50 Da). These analogues display tissue- and phenotype-specific patterns: for instance, analog +50 correlates with cardiac-variant disease, while analogues +16 and +34 associate with renal or neurologic involvement [47]. Quantification of the sum of urinary lyso-Gb3 + analogues has shown near-100% sensitivity and specificity for FD diagnosis, outperforming isolated lyso-Gb3 in detecting late-onset and female cases. A comprehensive glycosphingolipidomic approach was applied in a single-center cohort study using a multiplex UPLC–LC–MS/MS assay to quantify multiple glycosphingolipid species upstream and downstream of the enzymatic defect in Fabry disease. The study included 64 Fabry patients, comprising 27 symptomatic males (median age 47 years, all receiving enzyme replacement therapy) and 37 females, further stratified into asymptomatic females (median age 36.5 years, mostly not on ERT) and symptomatic females (median age 51 years, mostly on ERT). Control samples were obtained from 64 healthy individuals for plasma analysis and 12 for urine analysis, reflecting the challenges inherent to biomarker validation in rare diseases.

Beyond confirming plasma lyso-Gb3 as a robust marker of disease burden and progression, this study identified long-chain urinary ceramide dihexoside (CDH) isoforms as highly discriminatory biomarkers, particularly in female patients. Several CDH species (including C22–C26 isoforms) were significantly elevated in Fabry disease and showed stronger discrimination of asymptomatic heterozygous females than lyso-Gb3 alone (*p* < 0.001 vs. *p* < 0.01, respectively). These findings highlight that, despite relatively small cohort sizes, advanced lipidomic profiling can uncover complementary biomarkers with potential clinical utility, especially in subgroups where diagnosis remains challenging. However, the authors also emphasize the need for independent validation in larger, multicenter cohorts to confirm reproducibility and clinical applicability [48]. Additionally, the relative abundance of Gb2 and methylated Gb3 isoforms in urine may discriminate pathogenic variants from benign polymorphisms, suggesting their utility in newborn screening or genotype reclassification [49].

### 6.3. Accumulation vs. Response Biomarkers

Carnicer-Cáceres et al. introduced a distinction between accumulation biomarkers—directly reflecting glycosphingolipid storage (e.g., Gb3, lyso-Gb3, analogues, Gb2, CD77 expression)—and response biomarkers, which mirror the cellular and inflammatory reactions to substrate overload. The latter group captures secondary injury pathways (oxidative stress, inflammation, fibrosis, apoptosis) and may offer earlier detection of organ damage before irreversible structural alterations occur [46].

Among response biomarkers, plasma nitrotyrosine (3-NT), malondialdehyde (MDA), myeloperoxidase (MPO), glutathione peroxidase (GPx), and thiobarbituric acid reactive substances (TBARS) have been linked to oxidative stress and vascular injury. Increased sICAM-1, sVCAM-1, IL-6, TNF-α, and P-selectin levels confirm endothelial activation and metaflammation [50]. Importantly, these markers remain elevated even in ERT-treated patients, suggesting partial therapeutic uncoupling between enzyme correction and inflammatory tone.

### 6.4. Organ-Specific Biomarkers

FD’s multisystemic nature necessitates organ-focused biomarker panels:

- Renal involvement: Beyond albuminuria and estimated glomerular-filtration rate, podocyturia, urinary CD80, and urokinase-type plasminogen activator receptor (uPAR) have emerged as sensitive indicators of early podocyte injury [40]. Lyso-Gb3 exposure in cultured podocytes upregulates TGF-β1, Notch-1, fibronectin, and collagen IV, mediators of epithelial–mesenchymal transition and fibrosis [51]. Quantification of urinary podocin and podocalyxin by LC-MS/MS provides a reproducible, non-invasive readout of nephropathy onset [52].
- Cardiac involvement: High-sensitivity cardiac troponin T (hs-cTnT) and NT-proBNP remain the most validated markers of myocardial stress and fibrosis. Their increase often precedes imaging evidence of late-gadolinium enhancement. Carnicer-Cáceres et al. also highlight TGF-β1, VEGF, VEGFR2, FGF-2, MMP-2, and thrombospondin-1 (TSP-1) as potential cardiac remodeling mediators and surrogate biomarkers [46,53].
- Vascular and systemic inflammation: Alonso-Núñez et al. identified a circulating inflammatory–cardiovascular biomarker panel comprising TNF-α, MCP-1, MIP-1β, VEGF-A, ADAMTS-13, GDF-15, MPO, and MIC-1. Elevated levels correlated with disease severity, cardiac hypertrophy, and reduced renal function, suggesting prognostic and therapeutic-monitoring value. Notably, ADAMTS-13 deficiency—previously associated with endothelial dysfunction—was linked to enhanced microvascular injury and inflammatory activation in FD [8,54].

### 6.5. Omic and Molecular Biomarkers

Advances in omics technologies have revealed multi-layered molecular perturbations in Fabry disease (FD). Proteomic analyses of plasma and urinary exosomes have identified dysregulation of lysosomal proteins (e.g., cathepsins B and D), oxidative stress–related enzymes (SOD2, peroxiredoxins), and mitochondrial metabolic regulators, including ATP synthase subunits and cytochrome c oxidase components [44,48]. In parallel, transcriptomic studies in peripheral blood mononuclear cells have demonstrated activation of NF-κB, mTOR, autophagy, and endoplasmic reticulum stress pathways, with expression profiles correlating with cardiac and renal involvement [44,55].

MicroRNAs (miRNAs) are emerging as non-invasive and dynamic molecular biomarkers. Altered expression of miR-21, miR-29, miR-1307-5p, and miR-199a-5p has been associated with fibrosis, endothelial activation, and mitochondrial dysfunction in FD, suggesting a potential role in monitoring disease activity and therapeutic response [56,57]. However, despite their strong biological plausibility, most omics-derived biomarkers are currently supported by exploratory or small-scale studies, and their clinical applicability remains limited by heterogeneity, lack of standardization, and the need for large prospective validation cohorts. At present, these approaches should therefore be regarded as complementary research tools rather than clinically established biomarkers.

### 6.6. Imaging and Composite Biomarkers

Quantitative cardiac MRI (native T1 mapping, extracellular-volume fraction, late-gadolinium enhancement) and renal MRI (diffusion and perfusion metrics) are increasingly integrated as imaging biomarkers complementing biochemical data [58]. Cerebral MRI parameters—white-matter hyperintensity volume, basilar-artery diameter—also provide quantifiable indices of neurologic involvement [58]. Composite scoring systems such as the Fabry Stabilization Index (FASTEX) and Modified Mainz Severity Score Index (MSSI) combine biochemical, imaging, and clinical parameters to longitudinally assess disease control [59].

It should be noted that, while biomarkers such as lyso-Gb3 and cardiac imaging parameters are clinically established and routinely used for diagnosis and monitoring, several emerging molecular and inflammatory biomarkers should currently be regarded as exploratory or early translational tools, requiring further clinical validation.

## 7. Therapeutic Strategies and Novel Targets

The therapeutic management of Anderson–Fabry disease (FD) has evolved substantially over the past two decades, yet significant challenges persist in halting organ progression and reversing tissue remodeling. Traditional approaches have largely focused on replacing or stabilizing the deficient enzyme, while novel molecular targets are emerging from the expanding understanding of the disease’s inflammatory, metabolic, and mitochondrial underpinnings [60]. Specific and emerging therapeutic approaches in Fabry disease are summarized in Table 3.

### 7.1. Specific Disease-Modifying Therapies: Enzymes, Chaperones, and Gene Therapy

Enzyme replacement therapy (ERT) remains the mainstay of treatment. Recombinant α-galactosidase A—either agalsidase alfa, agalsidase beta, or the newer PEGylated form pegunigalsidase alfa—can reduce plasma and tissue Gb3 and lyso-Gb3, alleviate neuropathic and cardiac symptoms, and stabilize renal function [60]. Despite these benefits, ERT is limited by its short half-life, incomplete biodistribution to critical organs (particularly heart and brain), and the formation of neutralizing anti-drug antibodies.

Pharmacological chaperone therapy, using small molecules such as migalastat, selectively binds to specific amenable GLA variants, stabilizing misfolded α-galactosidase A and enhancing its trafficking to lysosomes [61].

The clinical efficacy and safety of migalastat have been evaluated in a randomized, double-blind, placebo-controlled phase III trial (AT1001-011), followed by an open-label extension (AT1001-041). The study enrolled adult and adolescent patients (16–74 years) with genetically confirmed Fabry disease carrying amenable GLA mutations, preserved renal function (eGFR > 30 mL/min/1.73 m^2^), and elevated urinary GL-3 levels. A total of 67 patients were randomized to receive migalastat (150 mg every other day) or placebo for 6 months, followed by open-label migalastat treatment for up to 24 months.

The primary endpoint was the reduction in GL-3 inclusions in renal interstitial capillaries, while secondary endpoints included urinary GL-3 levels, renal function, proteinuria, and safety. Tertiary outcomes comprised cardiac parameters, patient-reported outcomes, and α-galactosidase A activity. Migalastat demonstrated stabilization of renal function and substrate burden in patients with amenable mutations, supporting its disease-modifying potential in this subgroup.

Regarding safety, it was generally well tolerated. The most frequently reported adverse events were headache and nasopharyngitis, with a similar incidence compared to placebo during the double-blind phase. Most adverse events were mild or moderate, and no deaths or treatment discontinuations due to drug-related adverse events were reported. Serious adverse events were infrequent and rarely considered related to migalastat. Importantly, no progression to end-stage renal disease, cardiac death, or stroke was observed during the study period. These findings indicate a favorable safety profile and support migalastat as a clinically established oral therapy for Fabry patients with amenable GLA mutations.

Gene-therapy and mRNA-based strategies are now entering clinical stages. AAV-mediated liver-directed gene transfer and lipid-nanoparticle-encapsulated mRNA formulations have shown sustained enzymatic activity and Gb3 clearance in preclinical and early-phase trials. Nevertheless, uncertainty persists regarding long-term expression, vector immunogenicity, and potential immune activation [62]. Thus, while these approaches correct the genetic defect, they do not directly address the secondary molecular cascades—inflammation, oxidative stress, mitochondrial dysfunction, and fibrosis—that drive irreversible organ damage.

### 7.2. Pathway-Driven Therapeutics: Metabolic, Immune–Inflammatory, and Next-Generation Targets

Recent pathophysiological insights reveal that lysosomal substrate overload impairs autophagy and mitophagy, leading to the accumulation of dysfunctional mitochondria and excessive reactive oxygen species (ROS) production [70]. The resulting bioenergetic failure activates the AMPK–mTOR axis, disrupting cellular homeostasis. Pharmacologic activation of AMPK (e.g., by metformin, AICAR) and mTOR modulation have been proposed to restore autophagic flux and improve mitochondrial efficiency [70]. Experimental studies in fibroblasts and murine models suggest that mTOR inhibitors, such as rapamycin and everolimus, can normalize autophagy markers (LC3B-II, p62), potentially reducing Gb3 accumulation independently of α-galactosidase A activity [63,64].

In parallel, mitochondria-targeted antioxidants, including MitoQ, coenzyme Q10, and N-acetylcysteine, have shown the ability to reduce ROS production and improve endothelial function in preclinical FD models [70]. Strategies aimed at restoring mitochondrial biogenesis through PGC-1α activation (e.g., bezafibrate or resveratrol) may further counteract metabolic inflexibility and oxidative stress. However, these approaches remain largely investigational and are currently supported mainly by preclinical or proof-of-concept evidence [70].

Chronic inflammation represents another major therapeutic target in FD. Multiple studies, including those by Tuttolomondo et al. and Kurdi et al., emphasize that chronic inflammation is a central amplifier of tissue injury in FD. The TLR4/NF-κB pathway, activated by lyso-Gb3 and other glycosphingolipids, triggers transcription of pro-inflammatory cytokines (TNF-α, IL-1β, IL-6) and adhesion molecules (VCAM-1, ICAM-1) [65,66,67].

Preclinical evidence supporting TLR4 modulation as an anti-inflammatory strategy derives from seminal experimental studies by Shirey et al., demonstrating that genetic or pharmacological blockade of TLR4 signaling confers robust protection against inflammation-driven tissue injury. In murine models of severe influenza, TLR4-deficient mice were highly resistant to virus-induced lethality, while therapeutic administration of the synthetic TLR4 antagonist Eritoran (E5564) markedly reduced mortality, lung pathology, cytokine release, oxidative stress, and accumulation of oxidized phospholipids. Notably, Eritoran remained effective even when treatment was initiated several days after infection, indicating a capacity to interrupt self-sustaining inflammatory cascades rather than merely preventing early pathogen sensing.

Mechanistically, these effects were mediated by inhibition of TLR4 activation by endogenous danger-associated molecular patterns, including oxidized phosphatidylcholines generated through NADPH oxidase–dependent oxidative stress. Eritoran interfered with TLR4/MD2 signaling via CD14-dependent binding, thereby attenuating NF-κB activation, cytokine storm amplification, and downstream reactive oxygen species generation. These findings support a broader concept in which TLR4 functions as a central amplifier of inflammation driven by endogenous lipid mediators rather than by microbial ligands alone [66].

Although these studies were conducted in infectious and acute lung injury models, they provide a strong mechanistic rationale for targeting TLR4-driven “metaflammation” in Fabry disease, where lyso-Gb3, oxidative stress, and lipid-derived danger signals converge on TLR4/NF-κB pathways. However, it should be emphasized that TLR4 antagonists such as Eritoran have not yet been evaluated in Fabry disease, and current evidence remains limited to preclinical models. Therefore, TLR4 modulation should presently be regarded as a biologically compelling but early-stage translational strategy, requiring disease-specific validation before clinical application.

Pharmacological inhibition of TLR4 (e.g., TAK-242, Eritoran) or downstream NF-κB signaling has been shown in vitro to attenuate cytokine release and oxidative stress, although clinical data are currently lacking [67]. Similarly, activation of the NLRP3 inflammasome has been implicated in lysosome-dependent inflammation, linking Gb3 accumulation to IL-1β and IL-18 release and to vascular inflammation and fibrosis [68,71].

The complement system has also emerged as a potential therapeutic target. Uncontrolled activation of C3 and C5 fragments promotes microvascular inflammation and fibrosis. Early-phase studies are exploring complement inhibitors (e.g., eculizumab, ravulizumab, C5aR1 antagonists) as adjunctive therapies, particularly in antibody-positive patients receiving enzyme replacement therapy [72,73]. Complement-targeting agents, including eculizumab, ravulizumab, and C5aR1 antagonists, are currently not part of standard clinical care for Fabry disease. Their proposed use is based on indirect mechanistic rationale, observational findings, or extrapolation from other complement-mediated disorders, and remains investigational in the Fabry disease setting.

Finally, persistent inflammation and metabolic stress converge on fibrogenic pathways, mainly through TGF-β1, Notch-1, connective-tissue growth factor (CTGF), and matrix metalloproteinases (MMP-2, MMP-9) [71]. These mediators drive extracellular-matrix deposition and organ stiffening, particularly in the heart and kidneys. In FD-derived fibroblasts, TGF-β1 blockade using monoclonal antibodies or receptor-kinase inhibitors (SB431542) attenuates collagen synthesis and reduces SMAD-2/3 phosphorylation [71]. Angiotensin II receptor blockers (ARBs) and ACE inhibitors also inhibit TGF-β1 and NADPH oxidase activity, reducing fibrotic signaling. Emerging studies on MMP modulation suggest that balancing extracellular-matrix turnover may reverse early fibrotic remodeling. Agents such as doxycycline (a non-specific MMP inhibitor) or selective MMP-2/9 blockers are under evaluation for their potential to slow cardiac hypertrophy and interstitial fibrosis in FD [74].

MicroRNAs and epigenetic regulators represent additional next-generation targets: dysregulated miR-21, miR-29, miR-1307-5p, and miR-199a-5p have been linked to pro-fibrotic and inflammatory phenotypes, and experimental silencing of miR-21 reduces fibrosis in cardiomyocytes [75,76,77]. Epigenetic modulators, such as histone deacetylase inhibitors, may further enhance lysosomal biogenesis and autophagic efficiency, although their clinical relevance in FD remains to be established [78].

Overall, while these pathway-driven strategies are biologically compelling, most currently rely on preclinical or early translational evidence, and their clinical applicability will require rigorous validation in prospective human studies.

Beyond biochemical and organ-specific outcomes, emerging therapeutic strategies in Fabry disease may have relevant implications from a patient-centered perspective. Adjunctive approaches targeting metabolic dysfunction and chronic inflammation could potentially improve symptoms such as fatigue, pain, and exercise intolerance, which substantially affect quality of life but are not always adequately controlled by enzyme replacement therapy alone. In addition, oral treatments, including pharmacological chaperones and small-molecule modulators, may reduce treatment burden and improve long-term adherence compared with lifelong intravenous infusions. From the patient’s viewpoint, the possibility of personalized therapeutic strategies—based on genotype, inflammatory profile, or disease stage—represents a meaningful step toward more flexible and tolerable management of Fabry disease.

It should be emphasized that, with the exception of enzyme replacement therapy and pharmacological chaperones, most pathway-driven strategies discussed in this section are currently supported mainly by preclinical data or early translational evidence. Given the rarity of Fabry disease, available human studies are necessarily limited to small patient cohorts, and much of the encouraging evidence derives from experimental models or from mechanistically related conditions. To date, Fabry disease–specific randomized clinical trials are lacking for most metabolic, immune-inflammatory, or mitochondrial-targeted interventions. These approaches should therefore be regarded as hypothesis-generating adjuncts to conventional ERT or chaperone therapy, rather than as imminent clinical options for Fabry disease patients.

## 8. Discussion

Fabry disease emerges as a complex multisystem disorder in which lysosomal dysfunction, metabolic remodeling, organelle stress, and chronic inflammation form an integrated pathogenic network rather than isolated or sequential pathological events. The primary defect in α-galactosidase A leads to progressive accumulation of Gb3 and lyso-Gb3, disrupting lysosomal clearance, autophagy, and cellular metabolic homeostasis. These early molecular alterations propagate through interconnected pathways involving mitochondrial dysfunction, oxidative stress, endoplasmic reticulum stress, and immune activation, ultimately contributing to progressive cardiac, renal, and neurological injury. The persistence of these mechanisms even after partial biochemical correction underscores the limitations of a purely substrate-centered interpretation of Fabry disease.

Taken together, the evidence discussed in this review supports the view that Fabry disease should not be interpreted solely as a disorder of lysosomal storage or downstream organ damage, but rather as a complex network of interconnected molecular, metabolic, and immune–inflammatory disturbances. By focusing on the mechanistic crosstalk between lysosomal dysfunction, mitochondrial impairment, and chronic immune activation, the present work complements previous clinically oriented reviews and provides a conceptual framework aimed at deepening pathogenetic understanding rather than prioritizing therapeutic translation.

Clinical heterogeneity among Fabry patients likely reflects differences in residual enzyme activity, substrate burden, inflammatory signatures, and organ-specific vulnerability. Genotype–phenotype studies have demonstrated that distinct GLA variants are associated with divergent disease trajectories, reinforcing the need for personalized diagnostic and therapeutic strategies. Despite advances in enzyme replacement and chaperone therapies, many patients continue to exhibit progression of fibrosis, inflammation, and organ dysfunction, highlighting the pathogenic relevance of secondary mechanisms beyond lysosomal storage alone. A central insight emerging from recent research is the bidirectional interplay between metabolic and immune–inflammatory pathways.

Vascular involvement represents a fundamental component of Anderson–Fabry disease and contributes significantly to the multisystemic progression of the disorder. The accumulation of glycosphingolipids within endothelial and vascular smooth muscle cells promotes endothelial activation, oxidative stress, impaired nitric oxide bioavailability and progressive microvascular dysfunction, ultimately leading to tissue hypoperfusion and organ damage. In this framework, endothelial dysfunction and increased arterial stiffness represent early markers of vascular injury and have been shown to correlate with specific vascular phenotypes in ischemic stroke [79], supporting the hypothesis that microvascular impairment plays a central role in Fabry-related cerebrovascular involvement. In addition, blood pressure variability and impaired vascular adaptability, as assessed by 24-hour ambulatory monitoring, have been shown to influence vascular stress and endothelial function [80], mechanisms that may be particularly relevant in Fabry patients with autonomic dysfunction. These dynamic alterations may further exacerbate small-vessel vulnerability, contributing to structural and functional remodeling. Such vascular changes favor the development of white matter lesions, ischemic events and progressive cerebrovascular damage. Notably, the association between systemic fibrotic processes and cerebral white matter lesions has been observed in other chronic inflammatory–metabolic conditions [81], suggesting that shared mechanisms of endothelial dysfunction and extracellular matrix remodeling may also contribute to Fabry-related brain involvement. Overall, the interplay between endothelial dysfunction, autonomic imbalance, chronic inflammation and extracellular matrix remodeling reinforces the concept that Anderson–Fabry disease should be interpreted not only as a lysosomal storage disorder but also as a complex vascular and inflammatory condition.

These vascular alterations contribute to structural and functional remodeling of the small vessels, favoring the development of white matter lesions, ischemic events and progressive cerebrovascular damage. The association between systemic fibrotic processes and cerebral white matter lesions has been observed in other chronic inflammatory–metabolic conditions, suggesting that shared mechanisms of endothelial dysfunction and extracellular matrix remodeling may also contribute to Fabry-related brain involvement [80]. An additional layer of complexity relevant to precision-medicine approaches concerns the distinction between classic and late-onset Fabry disease phenotypes. Classic Fabry disease is typically associated with early and widespread glycosphingolipid accumulation, more pronounced systemic inflammatory activation, and higher circulating levels of lyso-Gb3 and pro-inflammatory mediators, mainly in male patients. In contrast, late-onset variants, often with a single organ involvement, exhibit lower systemic substrate burden but display evidence of mitochondrial dysfunction, oxidative stress, and localized immune activation within affected organs. These observations suggest that inflammatory and metabolic signatures may differ substantially across Fabry phenotypes, with important implications for biomarker stratification and therapeutic targeting. Recognizing such phenotypic heterogeneity supports the need for individualized diagnostic and therapeutic strategies rather than a uniform, substrate-centered approach.

Therapeutically, recognition of this interconnected pathogenic network supports a shift from enzyme-centric correction toward pathway-targeted modulation. Anti-inflammatory strategies targeting the TLR4/NF-κB and NLRP3 axes, antioxidants directed at mitochondrial ROS, and metabolic modulators of the AMPK–mTOR pathway represent rational adjuncts to enzyme-based therapies. Moreover, the involvement of profibrotic mediators such as TGF-β1, Notch-1, and matrix metalloproteinases highlights the importance of early intervention to prevent irreversible tissue remodeling. At a systems level, integration of multi-omics data and computational approaches may enable identification of patient-specific molecular signatures, supporting stratification beyond clinical staging.

## 9. Conclusions

In conclusion, this review proposes Fabry disease as a prototypical model of inherited metabolic inflammation, in which lysosomal storage, mitochondrial dysfunction, and immune dysregulation converge to drive disease progression. Rather than acting as linear or independent processes, these alterations form a tightly interconnected pathogenic continuum in which metabolic reprogramming, oxidative stress, and chronic inflammation mutually reinforce one another and promote irreversible organ damage.

By deliberately emphasizing the underlying biochemical and immunometabolic mechanisms—while addressing organ damage and therapeutic strategies as secondary consequences—this integrative framework shifts the focus from a storage-centered paradigm toward a deeper mechanistic understanding of disease biology. Such a perspective may support future advances in biomarker stratification and the development of precision-medicine approaches aimed at modulating metabolic inflammation as a central driver of Fabry disease progression. Whether this integrated immunometabolic model can be translated into clinical decision-making will require dedicated longitudinal and interventional studies.

## Figures and Tables

**Table 1:** Molecular and metabolic mechanisms involved in Fabry disease pathogenesis.

Pathophysiological Domain	Key Mechanisms	Main Molecular Players	Experimental/Clinical Evidence	Clinical Implications
Lysosomal dysfunction	Gb3 and lyso-Gb3 accumulation	α-Gal A deficiency, Gb3, lyso-Gb3	Human biopsies, plasma biomarkers, in vitro models	Initiates multi-organ damage; target of ERT and chaperones
Autophagy impairment	Defective autophagic flux and mitophagy	LC3-II, p62, AMPK–mTOR axis	Cell models, animal studies	Accumulation of dysfunctional organelles
Mitochondrial dysfunction	Bioenergetic failure, altered β-oxidation	ETC complexes, PGC-1α, acylcarnitines	Proteomics, metabolomics, fibroblast models	Contributes to fatigue, oxidative stress, organ vulnerability
Oxidative stress	Excess ROS production	NADPH oxidase, SOD2, peroxiredoxins	Plasma markers, experimental models	Amplifies inflammation and fibrosis
ER stress/UPR	Protein misfolding response	PERK, ATF6, CHOP	Cellular and animal models	Links enzyme misfolding to inflammation

**Table 2:** Emerging biomarkers in Fabry disease: type, source, analytical method, and clinical significance.

Biomarker Class	Examples	Biological Matrix	Analytical Method	Clinical Significance	References
Glycosphingolipids	Lyso-Gb3, analogues	Plasma, urine and also dried blood spots (DBS)	LC-MS/MS	Diagnostic, disease activity Note: DBS may facilitate screening and longitudinal monitoring in settings where venipuncture/sample handling is limited.	[16,32,33]
Inflammatory	TNF-α, MCP-1, IL-6, GDF-15	Serum/plasma	ELISA	Prognosis, treatment monitoring	[41,42]
Oxidative/metabolic	MPO, nitrotyrosine, AOPP	Plasma	Spectrophotometry	Early stress marker	[10]
Fibrotic	TGF-β1, MMP-2/9	Serum	Immunoassay	Predicts organ fibrosis	[10,21]
microRNA	miR-21, miR-29, miR-1307-5p	PBMCs, serum	RT-qPCR	Reflects fibrosis and mitochondrial dysfunction	[33,43]

**Table 3:** Specific and emerging therapeutic approaches in Fabry disease.

Therapeutic Class	Representative Agents	Mechanistic Target	Expected Effect	Status	References
Enzyme replacement	Agalsidase alfa/beta, Pegunigalsidase alfa	Substrate clearance	Reduces Gb3/Lyso-Gb3	Approved	[60,61]
Chaperones	Migalastat	Mutant enzyme stabilization	Increases α-Gal A activity	Approved	[61]
Gene/mRNA therapy	AAV, LNP-mRNA	Gene correction	Sustained enzyme synthesis	Clinical trials	[62]
Metabolic modulators	Metformin, Rapamycin	AMPK/mTOR	Restores autophagy	Experimental	[63,64]
Anti-inflammatory	TLR4/NF-κB inhibitors, MCC950	Innate immunity	↓ Cytokines, IL-1β	Preclinical	[65,66,67]
Anti-fibrotic	TGF-β inhibitors, MMP modulators	ECM remodeling	↓ Fibrosis	Translational	[68,69]
Mitochondrial protection	CoQ10, MitoQ, Resveratrol	ROS control, biogenesis	↑ Energy, ↓ stress	Experimental	[61,62]

## Data Availability

No new data were created or analyzed in this study.
